# Diagnosis of Diseases of Steroid Hormone Production, Metabolism and Action

**DOI:** 10.4274/jcrpe.v1i5.209

**Published:** 2009-08-02

**Authors:** John W. Honour

**Affiliations:** 1 University College London Hospitals, London, England; john.honour@uclh.nhs.ukClinical Biochemistry, University College London Hospitals, 60 Whitfield Street, London W1T 4EU England

**Keywords:** Steroid, investigation, clinical findings

## Abstract

Biochemical tests have been the basis for investigations of disorders affecting steroid hormones. In recent years it has been possible however to study the genes that determine functional enzymes, cofactors, receptors, transcription factors and signaling systems that are involved in the process. Analyses of mutations are available as a diagnostic service for only a few of these genes although research laboratories may be able to provide a service. Both biochemical and genetic research have brought to light new disorders. Some genes for transcription factors involved in the development of the endocrine organs have also been identified and patients with defects in these processes have been found. This paper will review general aspects of adrenal disorders with emphasis on clinical and laboratory findings. As with all endocrine investigations there are few single measurements that provide a definitive answer to a diagnosis. Timing of samples in relation to age, gender and time of day needs to be considered.

**Conflict of interest:**None declared.

Biochemical tests have been the basis for investigations of disorders affecting steroid hormones. In recent years it has been possible however to study the genes that determine functional enzymes, cofactors, receptors, transcription factors and signaling systems that are involved in the process. Analyses of mutations are available as a diagnostic service for only a few of these genes although research laboratories may be able to provide a service. Both biochemical and genetic research have brought to light new disorders but for some patients a precise diagnosis may still not yet be achieved. Some genes for trancription factors involved in the development of the endocrine organs have also been identified and patients with defects in these processes have been found. Specific points about the steroid assays will be covered in a separate review. In general there is a lack of harmonisation of assays, an issue of concern which needs to be addressed. Where hormone concentrations are given in this review as action points  these should not be used without reference to local experience and reference ranges for the analytical method in use. Tandem mass spectrometry is likely to replace the immunoassay procedures for steroids now being widely used; reference values are in general lower than has been the case before now. As with all endocrine investigations, there are few single measurements that provide a definitive answer to a diagnosis. Timing of samples in relation to age, gender and time of day needs to be considered. Appropriate reference ranges are needed. The regulation of a steroid needs to be taken into account. Test protocols should be agreed with the laboratory and adhered to in clinical practice. The laboratory should be given information about other drugs in use and clinical status of the patient (eg hypotensive). Any result that is unexpected should be discussed with the laboratory to exclude a procedural anomaly. A patient should be assessed clinically and biochemically before genetic tests are performed. Genetic tests will confirm the basis of the condition and provide a basis for treatment and genetic counseling. In some cases functional studies are needed to determine the effect on protein activity. Further genetic diseases may come to light from research based on patients with unusual clinical signs.

## CORTISOL HYPERSECRETION

**Figure 1:**
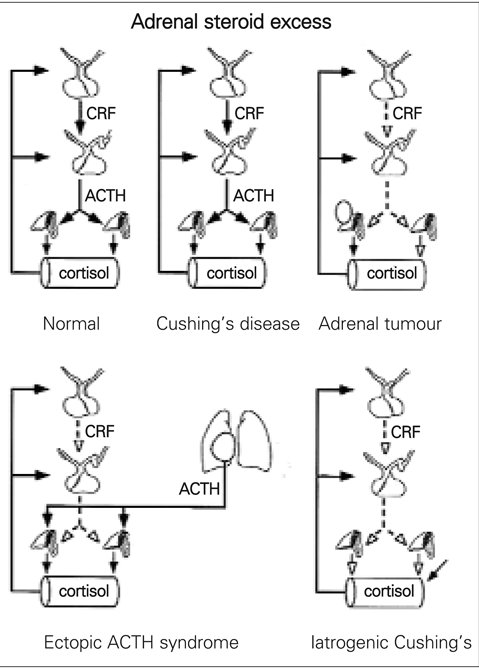
Adrenal steroid excess

High plasma concentrations of cortisol lead to combinations of a number of typical signs − moon−shaped face, buffalo hump, striae. The disease caused by a pituitary adenoma secreting adrenocorticotrophic hormone (ACTH) was first described by Cushing. This form is more frequent in boys than girls (1). Other causes of cortisol excess are classified under the generic name of Cushing’s syndrome (Figure 1). Use of exogenous corticosteroids is the commonest cause of such problems and this should be excluded before investigations are taken further. Growth retardation and weight gain are the more likely presentation in a child with Cushing’s syndrome. The high production of other steroids (more typical of adrenal tumours) accounts for acne, hirsutism, and in some cases for hypertension, although it is difficult to exclude direct effects of cortisol on blood pressure. 

Testing for Cushing’s syndrome first requires the demonstration of excess cortisol production and then establishing the degree of autonomy of this production (2, 3, 4). Total cortisol concentration can be raised if CBG is elevated, for example, with exogenous oestrogens. A raised 24 hour urine excretion rate of free cortisol is a good indicator for the condition. This is commonly requested in the investigation of an obese patient. In addition to the methodological considerations of quantifying free cortisol in urine, there are the problems of obtaining complete 24 hour urine collections, especially in children. A high cortisol excretion rate may reflect stress on the day of collection, but in children the effects of alcoholism and psychiatric disorders seen in adults are unlikely. 

Even if the urine cortisol excretion is clearly elevated (typically >400 nmol/24 hours), in the majority of patients tested, the urine free cortisol excretion will suppress to the lower region of the normal range after taking 0.5 mg of dexamethasone every 6 hours for 2 days. The urine collection is made on the second day of treatment. Children less than 40 kg should get a dose of 30 micrograms per kg per day (5). Serum or plasma cortisol concentrations can be measured at 0800−0900 h after taking 1.0mg of dexamethasone at 2300h as an alternative to this urine test. In children the dexamethasone dose is 0.3 mg per metre squared (6). Plasma cortisol results above 50 nmol/L are abnormal. Accelerated dexamethasone metabolism is however, seen in patients taking certain medications, particularly anticonvulsants. In this regard, the simultaneous measurement of cortisol and dexamethasone can improve the diagnostic precision of the test. In a patient with high clinical suspicion of Cushing’s, but normal urine free cortisol excretion rate on the first occassion, repeated 24h urine collections over many weeks may be necessary since cyclical forms of the  disease have been characterized, at least in adults,  in this way with peak frequency over  weeks to months.

The pattern of cortisol secretion in Cushing’s syndrome is such that there is a loss of the diurnal rhythm of the serum concentrations of cortisol with noteably high levels in samples taken at midnight. In adults saliva samples have been used, but this has not been validated in children (1, 7). Further biochemical tests and imaging procedures are justified at this stage. The protocol will vary between hospitals depending upon the laboratory services, clinical practice and experience, and according to the availability of specialised tests eg ACTH assay, CT scanning.

Measurements of the plasma ACTH concentrations at midnight and 0800 hours are invaluable in the distinction of Cushing’s syndrome. Circulating ACTH is low in patients with iatrogenic Cushing’s or an adrenal tumour. In the ectopic syndrome, the serum ACTH measured by RIA is often very high. In considering assays for ACTH, the specificity of the RIA or IRMA should be known with respect to the measurement of ACTH itself, POMC and proACTH. Theprecursors can be measured by relatively specific IRMA and are found at high concentrations in patients with ectopic tumours (8). Chromagranin A may prove to be useful in the differentiation of ectopic tumours (9).

If ACTH assays are not available, dynamic tests can be used to gain further indications for the pathology of a patient with high cortisol production. An adrenal tumour will not usually respond to an ACTH stimulation test with an increased steroid output, but a hypersecreting adrenal will respond with a significant rise in serum cortisol concentrations. In contrast to the ACTH stimulated adrenal which secretes largely cortisol, adrenal tumours produce a spectrum of steroids such as dehydroepiandrosterone (DHA) or 17−hydroxy pregnenolone or other steroids and measurements of these, specifically or generally, can be arranged through specialised centres. Analysis of steroids in urine using capillary column GC and with mass spectrometry (urinary steroid profile analysis) may thus be the simplest way to reveal complex patterns of steroid production associated with tumours.  This test an also confirm normal adrenal function should a mass be an incidentaloma. CT scanning may localise an adrenal tumour or display hyperplasia. Benign adrenal lesions associated with Cushing's syndrome are linked with dysregulation of cyclic AMP signaling, whereas tumours are linked to insulin−like growth factor II, p53 protein and related proteins (10).

The diagnosis of pituitary dependent Cushing’s disease is confirmed when previously high cortisol production is substantially suppressed with high doses of dexamethasone (2 mg 6 hourly for 2 days) (1). Plasma cortisol, ACTH and urine free cortisol (or better of total cortisol metabolites) should all fall to less than 50% of their basal values in the majority of affected patients. In a meta−analysis of 3 tests (UFC, dexamethasone suppression and midnight cortisol alone and in combination) was effective in the diagnosis of Cushing's syndrome (11). Up to 10% of patients with ectopic ACTH secreting tumours can, however, suppress with the high dose of dexamethasone. The plasma concentrations of potassium and ACTH should therefore be checked since hypokalaemia and very high ACTH values by RIA are characteristic of the ectopic tumour. Other ectopic tumours such as carcinoid may not always be associated with hypokalaemia and ACTH may be only moderately raised. Hypokalaemia is found in a majority of cases with ectopic ACTH tumour. These cases are rare in children. The adrenals are enormous on CT and cortisol production is usually grossly elevated. A patient with an ectopic ACTH tumour may present with rapid onset weight loss, oedema and muscle weakness which is not the classical clinical picture of Cushing’s syndrome.  

The common failure to distinguish the pituitary dependent from the ectopic ACTH secreting tumour and fear of the consequences of incorrect surgery are the reasons for the interest in further tests. If a pituitary adenoma is suspected a CRF test may be informative in many cases (1). In Cushing’s disease there is often an increase in cortisol above the normal response to intravenous 100 mg CRF (1 mg per kg in children). Blood samples are taken at 15 min before the CRF then at 0, 15, 30, 45, 60, 90 and 120 minutes. The combination of high dose dexamethasone and the CRF test with measurement of serum cortisol is superior to either test alone in the differential diagnosis of Cushing’s syndrome. The interpretation of this test in children is confounded by severe obesity (12). Simultaneous measurements of ACTH and cortisol may improve the sensitivity and specificity of the test (8, 13). If an ectopic tumour is suspected, the pancreas and chest should be scanned by MRI, but it has to be said that the primary tumour is not always found (1). Venous catheter studies for the localisation of tumours producing ACTH may be valuable. The measurement of tumour markers such as CRF and calcitonin should be considered.

Sampling of blood bilaterally from inferior petrosal sinuses (BIPSS) for simultaneous assessment of ACTH concentrations is now used in older children in some specialised centres to differentiate Cushing’s disease from the syndrome and for lateralisation of microadenoma (14). An intriguing observation from these studies has been the parallel increased secretion of prolactin, growth hormone, TSH and glycoprotein a−subunit.  These findings may reflect changes in the vasculature or a paracrine effect of b−endorphin from the tumour on adjacent tissue.  

Primary nodular adrenocortical hyperplasia is an mportant cause of Cushing’s syndrome in children and is usually associated with multiple endocrine neoplasia or Carney complex (15, 16). A paradoxical increase in UFC in the second phase of the high dose dexamethasone suppression test can be diagnostic (17, 18). McCune−Albright syndrome presents with Cushing’s syndrome in children along with polyostotic fibrous dysplasia, café−au−lait skin pigmentation and precocious puberty (19). The syndrome is caused by activating mutations of the G protein alpha subunit (20).  

Cortisone reductase deficiency is a rare cause of increased cortisol production because cortisone is not reduced to cortisol in the periphery. Patients present with a polycystic ovary picture without weight gain. Adrenal androgen output is raised because of the adrenal hyperplasia from high ACTH stimulation. Mutations can be found in the HSD11B1 gene and hexose−6−phosphate dehydrogenase (H6PDH) which is involved in NADPH regeneration for the enzyme (21). A urine steroid profile shows higher excretions of 11−oxo than 11−hydroxy cortisol metabolites (high ratio of THE plus cortolones to THF, allo−THF and cortols). A cortisone acetate challenge test is recommended to provide evidence to support the defects (22). Cortisol generation is lower than normal in patients with reductase defect.

High plasma cortisol concentrations and production rate can be seen in familial glucocorticoid resistance. Patients display signs of androgen and mineralocorticoid excess without the glucocorticoid effects (23, 24). Teenagers with resistant hypertension can show biochemical features of cortisol resistance (25), but so far the genetic basis of this has not been proven.

## ADRENAL CORTICAL INSUFFICIENCY

Adrenal insufficiency may result from primary adrenal failure or secondary disease to impairment of the hypothalamic−pituitary−adrenal axis (HPA) (26) and is life−threatening (27, 28). Although Addison in 1855 described tuberculous destruction of the whole adrenal glands, today autoimmune adrenalitis which spares the adrenal medulla is more common. In children, adrenal function is commonly impaired after long−term treatment with corticosteroids such as for asthma. This is an important clinical problem requiring tests, although there is no agreement on which tests should be performed. Morning plasma cortisol, urine free cortisol and cortisol response to ACTH are often used. Urine free cortisol is, however, a very poor test of adrenal suppression and measurements of total cortisol metabolites is much better (29). Congenital adrenal hyperplasia (CAH) is a common cause of primary adrenal insufficiency that can be missed (28, 30, 31, 32). Newborn screening based on determinations of blood spot 17−hydroxyprogesterone concentrations should pick up the 21−hydroxylase deficiency. A rapid result is needed to return an abnormal result before the onset of an adrenal crisis (presenting usually 5−10 days after birth). Hyperkalaemia before hyponatraemia can be early signs of CAH, prolonged jaundice in a newborn infant may also be a sign of adrenal insufficiency. Other rare causes of adrenal insufficiency include infarction or haemorrhage and adrenal hypoplasia (particularly in newborn infants). Later adrenal destruction by metastases, sarcoidosis, histoplasmosis and amyloidosis may be found. Patients may have few or no symptoms of adrenal cortical insufficiency until they suffer a physical stress such as trauma, surgery or infection when they present with tiredness, weakness, lethargy, anorexia, nausea, weight loss, dizziness and hypoglycaemia. Pigmentation will sometimes be noted as a smokey brown coloration which affects the buccal mucosa (inside cheeks, gums and lips), on skin creases, scars, genitalia and areolae. This is a reflection of melanocyte stimulating hormone action which is a product of the proopiomelanocortin secreted by the pituitary along with ACTH. The patient will probably have postural hypotension with high plasma renin activity (PRA). The differential diagnosis of hypoglycaemia in childhood should include adrenal cortical insufficiency. This can be due to congenital adrenal hypoplasia (33) due to a primary defect in adrenal development or defects in ACTH synthesis, processing, release and response (familial glucocorticoid deficiency) (34).  

Adrenal insufficiency is seen in disorders of cortisol production. Congenital adrenal hyperplasia in children often presents with symptoms due to excess or deficiency of sex steroids. Defects of 21−hydroxylase, 11β−hydroxylase and 3b−hydroxysteroid dehydrogenase are found with decreasing frequency and will be considered in more detail later in the review. Recently, an apparent combined deficiency of 21−hydroxylase and 17−hydroxylase was attributed to a defect in a common electron transporter called cytochrome P450 oxidoreductase (POR; ORD) (35, 36, 37). This defect can be associated with the Antley−Bixler syndrome.  A raised 17−hydroxyprogesterone is suggestive of 21−hydroxylase, other steroids (progesterone and corticosterone) need to be checked if a POR defect is likely.  A urine steroid profile is again a valuable test to cover these possibilities (38, 39, 40). Congenital adrenal hyperplasia due to defects of cholesterol uptake and cholesterol side chain cleavage enzyme (CYP11A1) (41, 42) can be lethal at an early stage, but there are exceptions (43) some present with hypospadias.  The infants present as phenotypic females and have an adrenal crisis in the newborn period.  Cholesterol uptake into mitochondria requires Steroidogenic Acute regulatory protein (StAR) and many mutations have been found in the gene (44, 45). Analysis of the genes involved in CAH has been critically reviewed (32) and will not be described in detail here. CYP21A2 mutations in Turkish children were summarized in this Journal (46). Several of the genes are duplicated on a chromosome to give active and inactive pairs (CYP21A2 and pseudogene CYP21B2), enzymes of different function (11−hydroxylase for cortisol synthesis − CYP11B1 and for aldosterone synthesis − CYP11B2) and enzymes of different tissue expression (HSD3B1 in periphery and HSD3B2 in adrenals and gonads).  Care must be taken in the genetic tests to demonstrate mutations.  Restriction enzyme digests, Southern blotting, polymerase chain reactions, specific oligonucleotide hybridization techniques, sequencing have been used. 

A number of disorders of adrenal and gonadal development have been characterized in recent years. They present clinically and biochemically with inadequate or absence of adrenal and/or gonadal steroids. These include in boys defects of nuclear receptors DAX−1 and SF−1. Mutations in the NROB1 gene are found in the dosage sensitive sex reversal adrenal hypoplasia congenita on the X−chromosome, in the gene 1 (DAX−1) lead to neonatal adrenal insufficiency and failure to undergo puberty with hypogonadotrophic hypogonadism (47, 48, 49, 50, 51). Steroidogenic factor 1 (SF−1), a nuclear receptor, is expressed in adrenals and gonads, and mutations in the gene are found in some patients that present with adrenal failure and 46XY DSD (52, 53, 54).

The ACTH receptor gene (MC2R) has been found to have mutations in infants that present with glucocorticoid but not usually mineralocorticoid failure (55, 56). Mutations that inactivate proopiomelanocortin (POMC) have been described in children with adrenal insufficiency, early onset obesity and sometimes along with fair skin and red hair (57). Other pituitary transcription factor genes can be affected (PROP1, HESX1, OTX2, LHX4, SOX3) (see Reference 26 for recent summary), and others will come to light in the course of time.

Indications of Addison’s disease in young males should prompt consideration of adrenoleukodystrophy (58, 59). Elevated levels of very long chain fatty acids (VLCFA) are found in plasma.  Neurologic problems may or may not be present. The achalasia−Addisonian−alacrima syndrome (AAA), which appears to be autosomal recessive, is another example of combined adrenal and neurologic (autonomic) involvement (60). A gene for aladin on chromosome 12q3 is involved (61). Smith−Lemli−Opitz syndrome may present as ambiguous genitalia, and distinctive facial features, small head size (microcephaly). Malformations of the heart, lungs, kidneys, and GI tract are also common. Infants with Smith−Lemli−Opitz syndrome have weak muscle tone (hypotonia), experience feeding difficulties, and tend to grow more slowly than other infants. Most affected individuals have fused second and third toes (syndactyly), and some have extra fingers or toes (polydactyly). A defect in cholesterol synthesis has been found and delta−7 steroids/sterols can be elevated in blood and urine (62, 63). 

A good algorithm for the investigation of primary adrenal insufficiency in children has been published from the Department of Pediatrics in Montreal, Canada (30).  Plasma cortisol concentrations between 7 and 9 am that are repeatedly less than 170 nmol/L are suggestive of adrenal cortical insufficiency. In a sick child cortisol concentrations of 300 nmol/L that do not increase on synacthen should be regarded as innappropriately low and worth investigating.  This may not be seen, however, until the course of the disease has advanced.  Low concentrations of cortisol in saliva also reflect Addison’s disease (64). A short Synacthen test should be performed to assess adrenal reserve.  Blood for serum cortisol is taken before and at 30 and 60 minutes after an intravenous injection of 250 micrograms of soluble Synacthen. Lower doses of synacthen (62.5 or 125 micrograms) should be used in young children. A normal response is characterised by an increment in cortisol of at least 200 nmol/L or a rise to levels above 500 nmol/L. If an assay for cortisol is specific enough to exclude cross reaction with prednisolone this synthetic steroid can be given immediately after the basal blood has been taken so as to afford glucocorticoid cover without affecting the adrenal response to exogenous ACTH. If there is pigmentation, the patient has primary adrenal failure (Addison's disease). Plasma ACTH measurements will be raised in Addison's disease and normal or low in secondary adrenal failure.  A metyrapone test or insulin tolerance test can be helpful in distinguishing primary from secondary adrenal insufficiency. Circulating antibodies to the adrenal cortex suggest an autoimmune process and other endocrine tests may be required to look for an extension of the autoimmune process to other hormonal tissues. Cortisol should be replaced at 9−12 mg/M^2^/24h and it is useful to check cortisol concentrations throughout the day in plasma samples taken at 30 minute intervals over 2 hours after a morning dose of  hydrocortisone, then at 2 to 3 hour intervals throughout the day.  Depending on the frequency of sampling, cortisol concentrations above 700 nmol/L within an appropriate pattern reflects adequate replacement therapy.

Hyponatraemia is a frequent finding in primary adrenal insufficiency due to loss of mineralocorticoid steroids.  The low plasma sodium is associated with hyperkalaemia and a raised plasma urea.  In the urine there will be low potassium loss.  In some cases the adrenal insufficiency may be part of more general hypopituitarism. If hypocortisolism is due to ACTH deficiency resulting from pituitary or hypothalamic disease such as tumours, infarction, trauma, there are usually other signs of deficiency of other hormones (eg loss of body hair). There will be no pigmentation. In the absence of ACTH, the production of aldosterone under the stimulus of the renin−angiotensin system will usually be unaffected and the blood pressure will probably be normal. Secondary adrenal insufficiency occurs when the adrenal cortex is deprived of ACTH stimulation. The commonest cause is after steroid therapy.  An expanding pituitary tumour which leads to impairment of pituitary function usually affects the pituitary hormones in a sequential manner over a period of time.  Growth hormone secretion fails first, followed by gonadotrophins, TSH and ACTH (65).

## MINERALOCORTICOID EXCESS

Primary aldosteronism is extremely rare in children (66). Increased aldosterone secretion may be suspected in a patient presenting with muscle weakness due to hypokalaemia and headaches due to hypertension. Before the laboratory enters investigations of the renin−angiotensin−aldosterone system, abnormalities of electrolytes and water balance need to be confirmed. Many anti−hypertensive drugs affect plasma renin and aldosterone (thiazide and loop diuretics, beta−receptor blockers, calcium channel blockers) and these should ideally be stopped two weeks before meaningful tests can be carried out.  Conn was the first in 1956 to describe an adrenal adenoma secreting aldosterone. The diagnosis of primary hyperaldosteronism rests in finding plasma aldosterone concentrations high for the corresponding PRA. Since removal of an adenoma corrects the blood pressure, localisation of the source of aldosterone assists a decision on the surgical approach for the operation. 

Blood samples must be taken on several occasions for measurement of electrolytes.  Patients with primary hyperaldosteronism have in the past been considered for investigation only when repeated plasma potassium values were below 3.7 mmol/L. This is now not the case. Causes for potassium depletion (diarrhoea, purgatives, liquorice ingestion, diuretics) must be excluded. An algorithm for the diagnosis of primary aldosteronism in adults can be followed to reach a diagnosis of a rare paediatric problem (67). The tests should be conducted under medical supervision. Not all tests have been validated in children and may be difficult. A tumour may accumulate radiolabelled cholesterol. Catheterisation of the adrenal veins is in some centres a successful procedure in adults, but the venous drainage of the adrenal glands is often complex and even an experienced radiographer is not guaranteed of success. In a patient with an aldosterone secreting adenoma, the adrenal venous serum from the affected side usually shows a higher concentration of aldosterone compared with either the contralateral adrenal vein or the periphery sampled simultaneously. Aldosterone and cortisol should be measured in all samples to check the authenticity of the site from which the blood is taken. The cortisol concentrations in the adrenal vein samples may be in the order of 6000 nmol/L. In children, idiopathic hyperaldosteronism in which the adrenals have a micro−or macronodular hyperplastic appearance has been described (68, 69). Surgery is not curative. The best treatment is with the aldosterone antagonist, spironolactone, or with amiloride. 

Glucocorticoid remediable hyperaldosteronism is a rare familial cause of hypertension in which the biochemical features of hyperaldosteronism and the hypertension respond to glucocorticoid (dexamethasone) treatment (70). The hyperaldosteronism is responsive to ACTH but not to angiotensin II. This condition is associated with increased excretion of 18−oxocortisol which may arise by 18−hydroxylation of cortisol in the adrenal zona fasciculata due to a protein from a chimeric gene of CYPB11B1 and CYP11B2 (71). Estimation of 18−hydroxycortisol is likely to prove useful in screening for this disorder (72, 73), but this assay has limited availability. An increased urine excretion of the steroid can be seen in a urine steroid profile. Genetic testing is available (74).

Diuretic therapy is the commonest cause of secondary aldosteronism. PRA is raised as a physiological response to hypovolaemia (haemorrhage or intestinal fluid loss). Renin secretion is increased with renal ischaemia, renal artery stenosis. Peripheral PRA levels may be only moderately raised, but renal vein samples may show a result on the ischaemic side 1.5 times or more that from the contralateral normal kidney.

Hypokalaemia without hypertension is the result of increased aldosterone, but certainly in adults can be due to diuretic or laxative abuse or psychogenic vomiting. Salt loss with hypokalaemic alkalosis and hypercaliuria are found with renal tubulopathies (Bartter syndrome) in the distal convoluted tubules and/or loop of Henle  (75). An infant with polyhydramnios may have one form of Bartter’s syndrome needing genetic analysis to distinguish them (76). Hypokalaemic alkalosis is due to a renal tubular defect from a mutation in the renal outer medullary potassium channel (Type 2 ROMK now KCNJ1) or the sodium/potassium/2 chloride transporter (Type 1 SLCl2A1 now NKCC2). The infantile form is associated with growth failure and sensorineural deafness. The primary cause for this disorder (type 3) is due to mutations in the chloride channel gene (CLCNKB) (76). Type 4 has simultaneous mutations in BSND and CLCNKB or CLCNKA. There is resistance of the vasculature to the pressor action of angiotensin II associated with a compensatory increase in PRA with juxtaglomerular hyperplasia. The plasma concentrations of both aldosterone and PRA are raised. The Gitelman variant can present in childhood with growth delay, metabolic alkalosis, hypokalaemia and hypomagnesaemia. Mutations are found in the chloride transporter SLCl2A3 gene (77).

Low PRA may be encountered when mineralocorticoids other than aldosterone are in excess. This can be due to CAH due to 11β−hydroxylase deficiency or 17α−hydroxylase deficiency (11−deoxycorticosterone DOC excess) (31, 32, 78) or with mineralocorticoid secreting tumours. All are rare. A defect in CYP11B1 presents with ambiguous genitalia in girls and precocious puberty in boys (79). 17−hydroxylase deficiency is not usually detected until a female is investigated for primary amenorrhoea and lack of sexual development. Persistent high progesterone is a useful diagnostic clue to this defect (80). The increased plasma corticosterone concentrations have been demonstrated by HPLC with UV detection (81). In a syndrome of apparent mineralocorticoid excess (AME) there is hypertension, hypokalaemia and reduced secretion rate of cortisol.  The improvement brought about by treatment with spironolactone (aldosterone antagonist) or triamterine (potassium sparing diuretic) suggested the presence of an unidentified mineralocorticoid. The disease is attributed to cortisol acting as both glucocorticoid and mineralocorticoid with a prolonged half−life due to low activity of 11β−hydroxysteroid dehydrogenase type 2 (which normally oxidises cortisol to  inactive cortisone) (82). This defect is most easily detected by a urine steroid profile which clearly displays a high excretion of cortisol metabolites relative to cortisone (83). Tandem mass spectrometry methods have enabled simultaneous analysis of cortisol and cortisone in blood (84, 85, 86) and saliva (87), and of metabolites in urine (88). The disease has been found mainly in children with severe hypertension and may be lethal which may explain why very few adult cases have been described. The mineralocorticoid activity of liquorice is now known to be due to inhibition of the enzyme system 11β−hydroxysteroid dehydrogenase. Patients should be asked about confectionary habits (89). 

Primary hypoaldosteronism with hyperkalaemia, increased PRA and low aldosterone concentrations is due to Addison’s disease, congenital adrenal hypoplasia, or defects of aldosterone synthesis most commonly due to CAH. PRA and aldosterone are rarely subnormal, but this secondary hypoaldosteronism is found in association with diabetes, chronic renal disease and as an isolated occurrence.

Hyponatraemia in the neonatal period is an urgent diagnostic problem which should consider whether the sodium intake is adequate (<4 mmol/kg/day in term babies, up to 12 mmol/kg/day in preterm infants), the water intake is high or there is sodium loss from the gastrointestinal tract or kidneys. Renal salt loss can be due to anatomical abnormalities, obstructive or renal tubular disorders which can include failure to respond to aldosterone. Low production of mineralocorticoid due to adrenal disease is a common cause of salt loss with hyperkalaemia in newborns.

A male child who collapses during the second week of life should be tested for CAH due to 21−hydroxylase deficiency. Sixty percent of all children with this defect show this salt−losing variant of the disease. The enzyme deficiency in cortisol production extends to the synthesis of aldosterone. Plasma renin activity will be elevated and aldosterone will be inappropriately low. Since plasma renin activity is higher in all newborn infants than in adults it is important to check the activity against a normal range for age of the infant (90). A defect in 3β−hydroxysteroid dehydrogenase, side−chain cleavage, StAR or adrenal hypoplasia may also explain the salt−losing state so other tests such as a urine steroid profile should be organized. 

If the biosynthetic path to cortisol is normal, the production of aldosterone needs to be evaluated. Defects of aldosterone production and action are recognised. In both cases PRA will be elevated, the defects are distinguished by the serum concentrations of aldosterone or urine excretion rates of the metabolites. A defect in the late steps of aldosterone biosynthesis will be confirmed when 18−hydroxycorticosterone production is shown to be elevated in blood (91) or urine (92). Mutations have been found in CYP11B2 by sequencing (93).       

Corticosterone, 18−hydroxycorticosterone and aldosterone are elevated through renin/angiotensin stimulation in disorders of aldosterone receptor and the gene NR3C2 encoding the mineralocorticoid receptor can have mutations in  some (pseudohypoaldosteronism − PHA) (94, 95, 96, 97). This is an autosomal dominant form of PHA1. A more common form is due to mutations of the epithelial sodium transport channel (ENaC) (98, 99). The biochemical diagnosis is straightforward if the child is sodium depleted (100). In children with the receptor defect the salt loss seems to be partially correctable by increasing the dietary salt intake to satisfy salt craving. Aldosterone receptor resistance has been demonstrated in assays of electrolyte transfer by mononuclear leucocytes (101), but this is not generally available.   

Hyponatraemia is often seen in the first weeks of life in preterm infants (<30 weeks gestation) (102). This reflects immaturity of renal function as well as in the adrenal production of aldosterone and the diuretic effect of increased vasopressin production (103, 104).

## ANDROGEN EXCESS

The investigations of androgen excess have different objectives depending on the age and karyotype of the patient. Thus 46,XX girls present with ambiguous genitalia usually due to inherited metabolic disease (congenital adrenal hyperplasia, CAH) of the adrenal cortex. During childhood boys with precocious puberty may have space occupying lesions of the brain, CAH or very rarely tumours of the adrenals or gonads. A few girls with hirsutism and/or acne with or without menstrual disturbance may have non−classic CAH or very rarely have excess secretion of androgens of ovarian or adrenal origin due to tumours. Polycystic ovaries are commonly found in mild hyperandrogenisation in adolescence that may progress to infertility in adult life.

**Figure 2:**
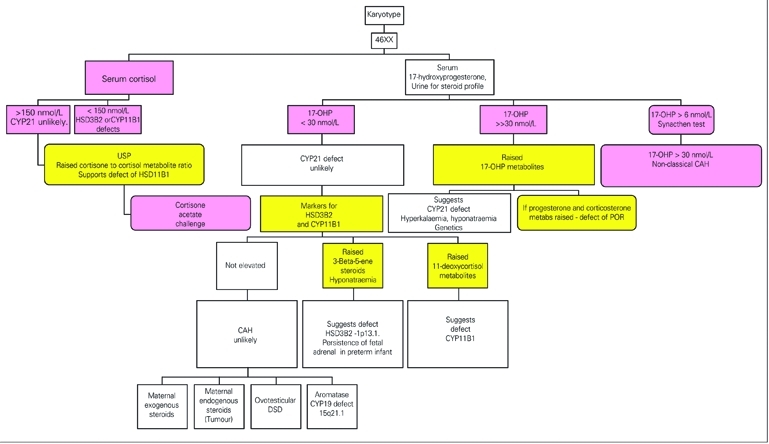
Androgenised female 46XX

In newborn girls with ambiguous genitalia (46XX DSD), the commonest cause is due to enzyme defects of cortisol synthesis with diversion of intermediates to androgen production (31, 32) (Figure 2). The genotype should be confirmed by chromosome analysis. A pelvic ultrasound may also be helpful, but results need to be treated with some caution. Other external causes of a virilised female are attributed to aromatase deficiency (105) and an ovotesticular DSD (106), maternal ingestion of progestogens (now very rare) or androgens or to maternal production of androgens by an adrenal or ovarian tumour. In these cases, the child will have normal endocrinology after birth, but may need surgery on the external genitalia if androgenisation is severe.

A reduction in steroid 21−hydroxylase activity or absence of 11β−hydroxylase or 3β−hydroxysteroid dehydrogenase can be the cause of CAH of which 21−hydroxylase deficiency is by far the commonest cause of CAH (90% of cases). In Europe, 60% of all cases of steroid 21−hydroxylase deficiency will present in the newborn period with a salt−losing crisis. This reflects the nature of the enzyme block. If there is a salt−losing crisis, this will need immediate treatment.  An increase in serum potassium may be seen prior to a fall in body weight and hyponatraemia.  

17α−hydroxyprogesterone is a biosynthetic precursor of cortisol and in patients with deficiency of 21−hydroxylase the production of 17−OHP increases and serum levels are elevated. The reduced capacity to produce cortisol, however, leads to high ACTH levels which cause adrenal hyperplasia. There is also a high adrenal secretion of androgens which cause virilisation. The measurement of 17−OHP in serum, plasma and blood spots is used for the diagnosis of this disorder. The timing of blood sampling is very important in order that the laboratory can interpret the findings. In all newborn infants, 17−OHP concentrations in serum are high on the first day of life (>100 nmol/L) and the levels fall over the first week to below 50 nmol/L. After day 3, there is usually good discrimination of the 17−OHP in affected cases (100−800nmol/L) from normal infants (<15 nmol/L). Differences in results with a direct and an extraction method would suggest that steroids in blood (probably steroid sulphates from the fetal adrenal) cross react in the RIA giving higher results in the direct assay (107, 108). The suggestion that steroid sulphates affect the quality of the assay is supported on the observation of greater discrepancy between the results obtained by the 2 methods in premature and low birth weight babies both of which have sustained fetal adrenal activity after birth. 17−OHP can be mildly elevated in defects of HSD3B2, POR and CYP11B1.

In rare cases of CAH, the defect is due to low activity of the 11β−hydroxylase enzyme (79). This defect is best identified by a raised serum concentration of 11−deoxycortisol or by a urine steroid profile. A high excretion of 6−hydroxy−tetrahydro−11−deoxycortisol (6−hydroxy−THS) is a better marker of the defect in the newborn than THS which is elevated in older patients (>120 nmol/L) but not so clearly raised in the newborn period (109). This again emphasises the requirement to involve specialised laboratories with relevant experience.

The cause of ambiguous genitalia in a newborn child is required as soon as possible in order to counsel anxious parents and start treatment. A 17−OHP assay which involves solvent extraction before the RIA is essential. Now that specific urine metabolites of 17−OHP have been recognised, the diagnosis by GC analysis of steroids in urine is reliable. Pregnanetriol is one marker for the disorder in the newborn. A characteristic urine steroid pattern can be recognised of which the most informative steroid is 17α−hydroxypregnanolone.  It is essential that a laboratory offering this analysis can provide a rapid service (<2 days). In order to achieve this, the identity of the steroids in the GC analysis must be confirmed by a further analysis with GC coupled to a mass spectrometer. Some laboratories now offer simultaneous analysis of several steroids by using GC−MS (110) or LC coupled with tandem mass spectrometry (111, 112, 113, 114). These tests enable one of several forms of CAH to be detected and will become important on regional or national basis. Newborn screening has been based on immunoassay of steroids in blood spots. Repeat testing has eliminated the disease when the concentrations of 17−OHP were lower than an initial high level that suggested 21−hydroxylase deficiency (115); this approach is stressful to the family. False positive results are reduced by the use of cut−off levels adjusted for gestation age or birth weight, and by solvent extraction of steroids (116). Screening will also improve with more specific technology based on tandem mass spectrometry (117, 118, 119).

Once CAH has been confirmed and lifelong cortisol treatment has commenced, compliance is best assessed in children by following growth (120). Height and weight should be followed at 3 monthly intervals in the first 2 years of life, then at 6 monthly visits. Bone age is checked yearly from X−rays of the wrist and hands. The replacement therapy should be adjusted according to body size. Hydrocortisone is usually prescribed at 9−12 mg/M^2^/day with 2/3 of the dose in the morning and 1/3 in the evening. If fludrocortisone is given, to control salt loss it must be remembered that this steroid is a potent glucocorticoid itself and the dose should not exceed 0.15 mg/M^2^. Sodium supplements may be needed in infancy. Electrolytes can be measured periodically, but for long term assessment of mineralocorticoid replacement the measurement of plasma renin activity is advisable. The measurement of 17−OHP and androgens in blood (or saliva) taken at regular intervals will define the adrenal steroid output in relation to treatment, but in practice has little effect on a patients drug taking habits. Androstenedione measurements may be helpful in the management of CAH due to 21−hydroxylase and 11β−hydroxylase defects. Measurement of PRA can be used to monitor efficacy of treatment in salt−losing CAH. Patients with 21−hydroxylase and some with 3β−hydroxysteroid dehydrogenase defects manifest elevated PRA, while in the defects with mineralocorticoid excess (17α−hydroxylase and 11β−hydroxylase) PRA is suppressed. PRA is normalised with effective treatment. In the case of 21−hydroxylase deficiency and 3β−hydroxysteroid dehydrogenase defects, treatment is improved with addition of fludrocortisone.

When sexual maturation appears before 8 years in girls and 9 years in boys, puberty is considered precocious. The diagnosis of precocious puberty was reviewed recently in this Journal (121). Normal puberty is initiated by an increase in pulsatile secretion of GnRH at night, although the mechanism and timing of this initiation is still not understood. Failure to find a pelvic mass by palpation or ultrasound scan reduces the likelihood of a rare ovarian tumour. Central precocious puberty (gonadotrophin dependent precocious puberty) reflects early activation of the gonadotrophic drive to increased gonadal function. This is more common in girls than in boys. A child with clinical precocious puberty who has pubertal gonadotrophin levels and augmented nocturnal gonadotrophin secretion has central precocious puberty (122, 123). LH results should be treated with caution because of interference from heterophilic antibodies and high results should be confirmed using a second assay (124). Assays have been devised to avoid such interference (125, 126). In girls, pelvic ultrasound is useful for the assessment of central precocious puberty (122). CNS tumours (hamartomas, third ventricular cysts, astrocytomas or gliomas) can be recognised by MRI. Some of the tumours (dysgerminomas, hepatoblastomas) secrete hCG. A cerebral tumour is relatively more common in boys with central precocious puberty than in girls.  

Gonadotrophin independent precocious puberty is due to inappropriate production of gonadal or adrenal hormones which affect secondary sex characteristics. Children may have acne and behavioural problems and may become taller than their peers. As a result of rapid skeletal maturation there is early epiphyseal fusion and the outcome is for short stature in adulthood. Hypothyroidism should be excluded. With the increase in TSH there is a concomitant increase in FSH and prolactin that presumably leads to sexual precocity. In patients with the McCune−Albright syndrome, precocious puberty is associated with cafe−au−lait skin marks and polyostotic fibrous dysplasia (19). Plasma gonadotrophin concentrations are often pubertal, but may in some cases be prepubertal.  Symptoms may wax and wane and on ultrasound this can be attributed to the appearance and regression of unilateral ovarian cysts. Up to 80% of girls and 40% of boys with precocious puberty may have no responsible cause and are termed idiopathic.  

Pseudo−precocious puberty can be the result of exposure to exogenous sex steroids. Both contraceptive pill and anabolic steroids are reported offenders. Abnormal sex steroid secretion from tumours is also a cause.  The most common adrenal tumour reported in the literature secretes DHAS. In general the tumours have been quite large. A number of cases of tumours secreting other androgens have been reported and using urine  steroid profile analysis, the secretion of 11β−hydroxyandrostenedione has  been defined on the basis of high excretion of 11β−hydroxyandrosterone (127, 128). In these reported cases androgen excretion was not grossly elevated and without scanning of the adrenals a tumour may have been dismissed. FSH and LH are suppressed to within prepubertal ranges. The secretion of androgens by adrenal tumours is not suppressed by giving dexamethasone. A testicular mass with grossly elevated 17−OH−P usually indicates CAH with adrenal rests in the testes. Leydig cell tumours, however, may produce elevated 17−OH−P, which is not suppressed with dexamethasone. 21−deoxycortisol is not raised in these cases, unlike the situation with CAH. One recent report of premature pubarche described a child with hyperandrogenic anovulation, who had low plasma DHAS due to defect of the sulphotransferase SULT2A1 through a defect in sulphate delivery from PAPS through inactivating mutations in PAPSS2 (129).

**Figure 3:**
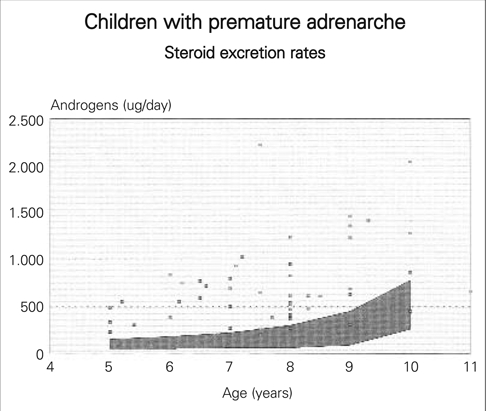
Children with premature adrenarche

Pubic hair growth before breast development or testicular enlargement may be the outcome of increased secretion of DHA and DHAS from the adrenal cortex due to early differentiation of the zona reticularis. This is a benign disorder called premature adrenarche not requiring treatment. DHA and DHAS concentrations in serum should be interpreted against normal ranges for age. Testosterone may be slightly elevated for age due to peripheral conversion of the adrenal androgens. The 24 hour urine excretion of androsterone, aetiocholanolone as well as cortisol metabolies are above the normal range for age and body size (Figure 3) reflecting advance adrenal growth. Premature adrenarche is more common in Asian and Afro−Caribbean children than Caucasians. 

Acne, hirsutism, menstrual disturbance can be due to non−classical forms of CAH and a mild defect of the steroid 21−hydroxylase. In many such patients, the basal 17−hydroxyprogesterone concentrations are above the normal range (>5 nmol/L). An injection of ACTH will lead to an increase in serum 17−OHP at 30 and 60 minutes after the trophic hormone injection and affected subjects will have an increment in 17−OHP greater than seen in normal subjects (Table 1) (130). Fifty percent of patients with non−classic or late onset form of CAH have HLA B14, DR1 in association, since this is coded nearby on the short arm of chromosome 6 and specific mutations in exon 1 or exon 7 CYP21A2 (V281L; P453S) (131). A late onset form of 3β−hydroxysteroid dehydrogenase deficiency was suggested on the basis of exagerated DHA or 17−hydroxypregnenolone  response to ACTH (132]), but mutation analysis does not support this aetiology (133, 134).

Some girls present with isolated breast development (premature thelarche) and no growth acceleration (135). Increased oestrogen production in girls with premature thelarche is a benign condition not to be confused with central precocious puberty. There is no increase in growth rate. The latter is more serious and causes progressive breast development with pubic hair growth, accelerated growth rate and bone maturation and early epiphyseal fusion. A pelvic ultrasound may show a follicle in the ovary. The two conditions have been resolved by repeated blood sampling at 15 minute intervals throughout the night (122). In patients with thelarche the FSH is higher (2−7 iu/L) than the LH (1−3 iu/L), which contrasts with precocious puberty where LH secretion predominates. A GnRH stimulation test may sometimes be needed to distinguish the 2 disorders again by revealing differences in the prodominant gonadotrophin, but interpretation may be assay dependent (136, 137). Various doses of GnRH are used with 10 microgram and 100 microgram (being the most common) with blood samples at zero, 20 and 60 minutes. Peak LH usually occurs at 20 min and peak FSH at 60 minutes after injection of GnRH.

**Table 1:**
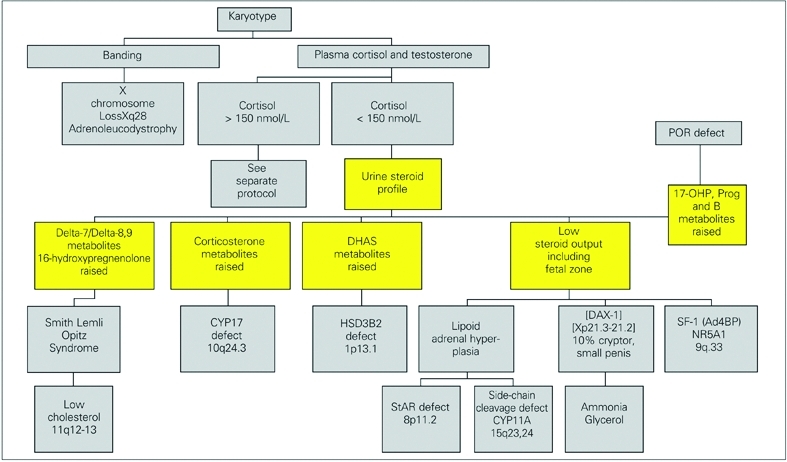
17−OHP by GC−MS − Reference ranges

## ANDROGEN DEFICIENCY

**Figure 4:**
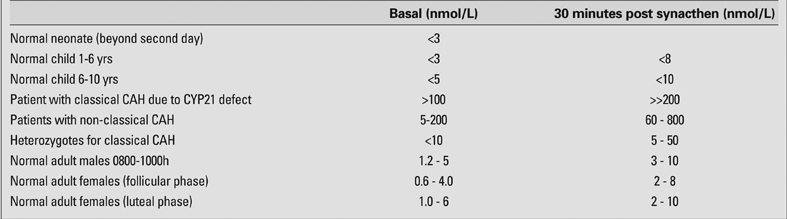
Poorly androgenised male, low cortisol (* can be ≥150 nmol/L unwell)

**Figure 5:**
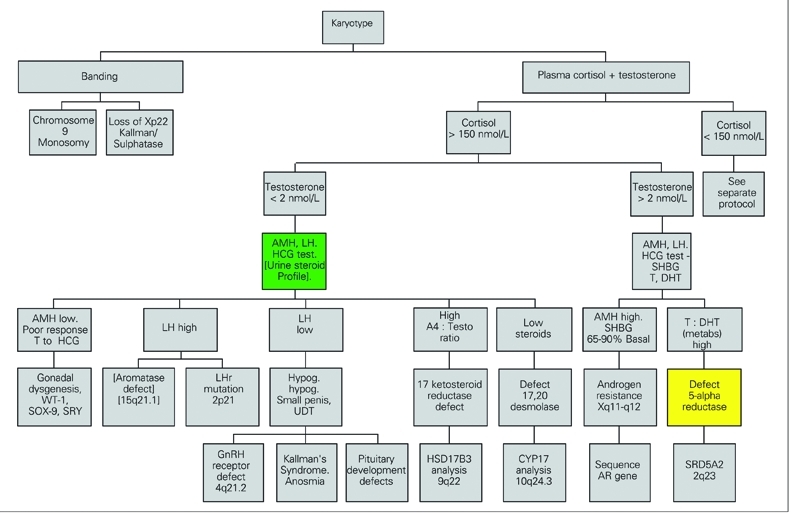
Poorly androgenised male, normal cortisol production (N.B. cortisol cut off can be > 300 nmol/L if unwell)

Incomplete androgenisation of a 46XY fetus is due to a failure of sex steroid production or to resistance to these hormones because of receptor defects. Most laboratories will need the help of specialised centres to resolve such cases, although a number of investigations can be undertaken locally. Following confirmation of chromosomes, the serum concentrations of cortisol and testosterone should be checked. From these results some clear decisions can be made. Defects of sex steroid production can be separated into:

• those affecting cortisol and androgens and (Figure 4)

• those relating to androgens alone (Figure 5)

Poor androgenisation of a 46XY male (46XY DSD) with low cortisol production can be attributed to defects of StAR, cholesterol 20,22 desmolase (side−chain cleavage, CYP11A1), 17α−hydroxylase deficiency, or of 3β−hydroxysteroid  dehydrogenase type 2 (Figure 4). These enzymes affect the production of all important adrenal and gonadal steroid hormones and disorders of the enzymes are often fatal for the child, so that few cases are documented in the literature. StAR deficiency is also called lipoid adrenal hyperplasia on account of the histological appearance of the adrenals at post mortem reflecting the ACTH stimulated adrenal which cannot produce cortisol nor process cholesterol.  

Defects of 17α−hydroxylase (78, 81, 138) and 3β−OH−S−DH are identified biochemically by demonstrating high serum levels and urine metabolites of the respective enzyme substrates (Table 1). The pattern of steroids in urine of a neonate, later confirmed to have 17α−hydroxylase deficiency, showed high excretion rates of 16α−hydroxypregnanolone. At 15 months of age the child excreted corticosterone metabolites just as is found in urine of adults with this disease (139). Mutations in CYP17 have been confirmed by sequencing (78, 81, 138, 140). A 3β−hydroxysteroid dehydrogenase deficiency is in practice difficult to confirm in a newborn child because the markers for the defect (DHA and pregnenolone) are normal products of the adrenal in the newborn due to inactivity of this enzyme in the fetal adrenal cortex. A 24 hour urine collection with determination of excretion rates of all steroids is therefore needed. Since these infants are usually very sick, this is not straightforward.  Plasma measurements of DHA and ACTH may be better tests before treatment, but in the face of an adrenal crisis replacement therapy may be needed before the necessary tests are completed. To overcome this problem dexamethasone is the preferred treatment. If a child suspected of having 3β−hydroxysteroid dehydrogenase deficiency is switched to treatment with dexamethasone and fludrocortisone then given daily injections of depot ACTH (Synacthen), the markers for the defect can be displayed in the urine steroid profile without interference from dexamethasone. This approach has been used successfully in confirming 3β−OHSDH deficiency during maintainance with hydrocortisone for several years, although ACTH was needed for several days before the suppressed adrenal secreted sufficient steroid to be detected in the urine steroid assay. Males with defects in HSD3B2 can be reared as boys needing adrenal steroid replacement with the addition of androgens at puberty. Mutations are located in HSD3B2 in regions coding for domains crucial for enzyme function (134, 141). Smith−Lemli Opitz syndrome is a dysmorphic disorder with microcephaly, short nose, pyloric stenosis and cleft palate. Cholesterol synthesis fails at the 7−dehydrocholesterol reduction step and 7−dehydro steroids can be found in the urine of an infant with this condition (142). 

The further investigation of males with poor androgenisation and normal cortisol will depend upon the initial findings for testosterone and gonadotrophins. A low basal testosterone with elevated gonadotrophins and a poor androgen response to HCG suggest either Leydig cell hypoplasia (LCH) or an androgen biosynthetic defect. Mutations in the LH receptor gene are responsible for LCH (143, 144). A high ratio of androstenedione to testosterone in the basal state, exagerated or revealed by HCG stimulation (ratio >2) suggests a defect of 17−ketosteroid reductase and genetic testing of 17BHSD3 may be needed (145, 146, 147). The immunoassays in use for androgens are not accurate in young children. A male with this defect can be reared as a boy but will need testosterone treatment. Improved, simultaneous androgen assays by tandem mass spectrometry will aid the diagnosis of DSD (148, 149, 150, 151).

A normal basal testosterone with a rise following HCG suggests that the problem is due to impaired action of testosterone. This can be due to a failure of target tissue 5α−reductase or to receptor defects. Specialist help will be needed to resolve this problem. A high testosterone to 5α−DHT ratio (>20) after HCG stimulation supports 5α−reductase deficiency. In a urine steroid profile there is evidence for this disorder also in the distribution of cortisol metabolites (5α−THF to THF) in childhood. In newborns, however, the majority of cortisol metabolites have been oxidised to cortisone metabolites and there is very little of the cortisol metabolites to effectively confirm this diagnosis. This test should therefore not be performed in the first six months. In that period, blood samples for androgens can be taken, because LH secretion is not suppressed (152). Missense and nonsense mutations in SRD5A2 have been reported (153, 154). Androgen receptor defects can influence the number and stability of androgen receptors. Genital skin needs to be taken and sent to a specialist laboratory. The cells have to be increased in number by culturing through several passages in tissue culture before these experiments can be undertaken. Receptor gene analysis is available in a few centres (155, 156, 157, 158, 159). Increased ratios of 5α to 5β reduced androgens have been found in association with Serkal syndrome (sex reversal with dysgenesis of kidneys, adrenals and lungs) possibly reflecting increased 5α−reductase and loss of function of WNT4 signalling (160).

Males with defects in androgen action may be considered for rearing as female. Mutations in SF−1 should be screened in cases of suspected partial androgen insensitivity when no mutations are found in the androgen receptor gene (161). Mutations in SF−1 have been found in boys with hypospadias or delayed puberty and no history of adrenal failure (162). Mutations in the gene for DAX−1 are found in boys that fail to undergo puberty with hypogonadotrophic hypogonadism and history of neonatal adrenal insufficiency without ambiguous genitalia (47, 48, 55). GnRH deficiency results from failure of embryonic migration of cells from the olfactory lobe to the forebrain. This is associated with anosmia in most cases (Kallman’s syndrome) and five genes have been implicated through mutation analysis of FGFR1, FGF8, PROKR2 and KAL 1 (163) and CHD7 (164). A mutation in the GnRH receptor gene has been described (165).

Inhibin B and AMH are respective markers of Leydig and Sertoli cell function. AMH concentrations are high in the first month of life (3.2−68.5 ng/ml) (166) and fall during childhood up to puberty (0.5−22.5 ng/ml). AMH is low in gonadal dysgenesis (167), partial androgen insensitivity syndrome and when there are mutations in the gene affecting AMH production (168). AMH is high when there are defects in testosterone synthesis and complete androgen insensitivity (167, 168). Inhibin B is at highest concentrations in infancy (100−500 nm/ml), low during childhood (<50 ng/ml) then rise in the early stages of puberty along with the increase in serum testosterone and testicular volume (169, 170). Basal AMH levels are high in androgen insensitivity syndrome (171).

Puberty is said to be delayed when there are no signs of puberty in boys by the sixteenth year and fourteenth year in girls (172). Complete absence of clinical signs in girls by the fifteenth year warrant investigation. The causes of delayed puberty are many. Gonadotrophin measurements will distinguish primary and secondary forms. The most common problem is isolated gonadotrophin deficiency; in short children this may be constitutional delay of normal puberty. The initial endocrine assessment of amenorrhoea includes measurements of prolactin, FSH, LH, oestradiol, TSH, and thyroxine. Hyperthyroidism is occassionally associated with amenorrhoea. When the serum prolactin exceeds 1000 iu/L, a prolactin secreting adenoma needs to be confirmed by appropriate scanning techniques. Hyperprolactinaemia is a cause of arrested puberty and successful medical or surgical treatment will restore regular cycles. Disordered ovarian function presents with primary amenhorroea. In some patients this can co−exist with signs of androgenisation. Body size and degree of exercise are important clinical considerations. Failure to show growth of the uterus on ultrasound also provides valuable diagnostic information. Failure to have a menstrual bleed following a progestogen challenge test is usually a further indication of low oestrogen production (poor follicular growth). A karyotype will exclude Turner syndrome and Turner mosaic. Low serum LH and FSH suggests hypogonadotrophic hypogonadism and these patients can respond well to pulsatile GnRH treatment. Inactivating mutations of the FSH receptor have been found in a few patients with hypergonadotrophic hypogonadism (173). When LH is raised in the presence of low or normal FSH, the most likely diagnosis is polycystic ovaries (rarely indicative of pregnancy due to reaction of HCG in the LH assay). Patients with PCO may have oligomenorrhoea and have a characteristic ovarian picture on ultrasound, and may have elevated LH concentrations such that the LH to FSH ratio is 3 or more.   

When primary amenorrhoea is associated with hirsutism, measurements of testosterone, DHAS, 17α−hydroxyprogesterone, cortisol and prolactin are required to identify or exclude major causes of  abnormal androgen production. Serum testosterone >5 nmol/L is indicative of a rare ovarian tumour. DHAS >20 mmol/L suggests the possibility of an adrenal androgen secreting tumour. 17α−hydroxyprogesterone >5 nmol/L is an indication of some degree of adrenal 21−hydroxylase defect. An ACTH stimulation test with exagerated response of 17OH−P is required to confirm this disease. The patient may be a heterozygote for the classical disease or may have a milder form sometimes referred to as late onset CAH. In very specialised laboratories 21−deoxycortisol is a better marker for a defective 21−hydroxylase. These patients can be treated with glucocorticoids.

Delayed puberty in boys requires similar investigations of the hypothalamic−pituitary−gonadal axis. Height and testicular volume should be measured. Serum testosterone shoud be measured early in the morning. Testicular failure can be due to torsion, surgery or inflammation. Gonadotrophin deficiency can be due to syndromes such as Kallman’s syndrome, A prolactinoma or pituitary hormone deficiency may be uncovered. A cranial MRI scan may be needed. Prader−Willi and Lawrence−Moon−Biedel. Kallman’s syndrome is associated with anosmia. During the last 20 years mutations in many genes in GnRH neuronal embryogenesis (KAL1, GnRHR, FGFR1, GPR54, PROK2, PROK2R, FGF8, CHD7, TAC3, TAC3R, NELF) (174, 175) and pituitary development (Prop1, HESX1, POUF1, LHX3, LHX4, SOX2, SOX3) (176, 177, 178, 179, 180, 181, 182) have been indentified.

**Table 1:**
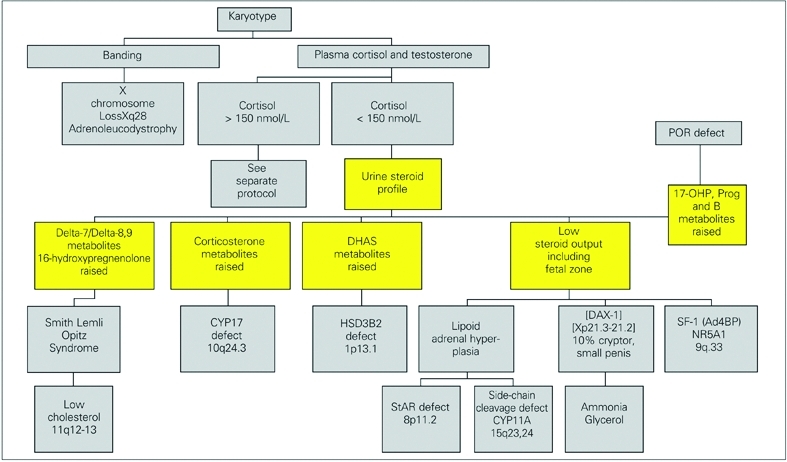
17−OHP by GC−MS − Reference ranges

## SUMMARY

This review has covered the clinical problems in children associated with steroid hormones. The excess or absence of cortisol, aldosterone and sex steroids were dealt with in turn so as to rationalise diagnostic tests.  The literature cited for reference includes many recent review articles. The laboratory will contribute to future improvements in assays with improved specificity and accuracy that will aid the differential diagnosis of related diseases, elucidate the causes of common clinical problems and further our understanding of developmental milestones such as adrenarche and puberty. The search for genes involved in endocrine function and development continues at a pace and new factors will come to light even between the time of writing this article and publication. This makes a review of this nature difficult to keep totally up to date.

## ACKNOWLEDGEMENT

I am grateful to John Achermann (Hospital for Children, Great Ormond Street, London, UK) for criticism, comment and advice on the draft manuscript.
